# Psychoactive drugs and male fertility: impacts and mechanisms

**DOI:** 10.1186/s12958-023-01098-2

**Published:** 2023-07-28

**Authors:** Moses Agbomhere Hamed, Victor Olukayode Ekundina, Roland Eghoghosoa Akhigbe

**Affiliations:** 1 Department of Medical Laboratory Science, Afe Babalola University, Ado-Ekiti, Ekiti State Nigeria; 2 The Brainwill Laboratory, Osogbo, Osun State Nigeria; 3 Reproductive Biology and Toxicology Research Laboratories, Oasis of Grace Hospital, Osogbo, Osun State Nigeria; 4 Department of Physiology, Ladoke Akintola University of Technology, Ogbomoso, Oyo State Nigeria

**Keywords:** Cannabis, Marijuana, Depressants, Drug abuse, Hallucinogens, Male infertility, Narcotics, Opioids, Psychoactive drugs, Stimulants

## Abstract

Although psychoactive drugs have their therapeutic values, they have been implicated in the pathogenesis of male infertility. This study highlights psychoactive drugs reported to impair male fertility, their impacts, and associated mechanisms. Published data from scholarly peer-reviewed journals were used for the present study. Papers were assessed through AJOL, DOAJ, Google Scholar, PubMed/PubMed Central, and Scopus using Medical Subjects Heading (MeSH) indexes and relevant keywords. Psychoactive drugs negatively affect male reproductive functions, including sexual urge, androgen synthesis, spermatogenesis, and sperm quality. These drugs directly induce testicular toxicity by promoting ROS-dependent testicular and sperm oxidative damage, inflammation, and apoptosis, and they also suppress the hypothalamic-pituitary–testicular axis. This results in the suppression of circulating androgen, impaired spermatogenesis, and reduced sperm quality. In conclusion, psychoactive drug abuse not only harms male sexual and erectile function as well as testicular functions, viz., testosterone concentration, spermatogenesis, and sperm quality, but it also alters testicular histoarchitecture through a cascade of events via multiple pathways. Therefore, offering adequate and effective measures against psychoactive drug-induced male infertility remains pertinent.

## Introduction

Psychoactive drugs are substances that alter the functions of the nervous system and result in the modulation of perception, mood, consciousness, cognition, and behaviour, including sexual behaviour. These drugs may have either licit (acceptable) or illicit (prohibited) usage. This heterogeneous class of drugs (Table 1) is commonly used for medical and recreational purposes. The use of psychoactive drugs without restraint is expanding rapidly per day, making the misuse of such drugs a public health concern [31, 70]), coupled with the need for an in-depth understanding of the pathophysiological impacts. Until recently, these brilliant tints and labelled substances were readily available via peddlers' outlets, often known as "drug stores," or the internet [31].

**Table 1:** Classification of psychoactive drugs

Stimulants	Depressants	Opioids (narcotics)	Hallucinogens
Cocaine	Benzodiazepines (e.g.rohypnol)	Heroine	Lysergic acid diethylamide (LSD)
Amphetamine	Diazepine	Codeine	Mescaline
Methamphetamine	Alcohol	Morpine	Psilocybin
Nicotine	Barbiturates	Opium	Ketamine
Caffeine	Gamma-hydroxybutyrate	Oxycordone	Ecstasy

Abusers of psychoactive drugs in a culture or group are easily identifiable by their unfavourable effects on both consumers of these substances and non-users [63]. The International Classification of Diseases, 10th Revision (ICD-10) classification takes into account the specific mental and behavioural problems that are associated with substances like alcohol, nicotine, opioids, cocaine, stimulants, hallucinogens, sedatives, hypnotics, cannabis, cannabinoids, and volatile solvents [32]. Ethical issues have prevented human interventional studies on the effects of smoking cigarettes, being around second-hand smoke, abusing recreational drugs, and drinking alcohol. As a result, observational studies constitute a chunk of the available data in the literature [43]. However, there are several reports on animal models [92].

Since substance abuse is on the rise globally, resulting in a global menace of public health concern, and psychoactive drugs seem to be a significant component of popularly abused drugs and cocktails, understanding the mechanistic effects of these drugs on male fertility is essential. Therefore, this narrative review focuses on psychoactive drugs' male reproductive health consequences. The associated (histo)pathological mechanisms are also discussed. The information provided in the present study will enhance our understanding of the pathogenesis of psychoactive drug-induced male infertility. It will help policymakers make decisions and open a window of therapeutic opportunities for managing psychoactive drug-induced male infertility.

## Cause of male fertility

**Fig. 1:**
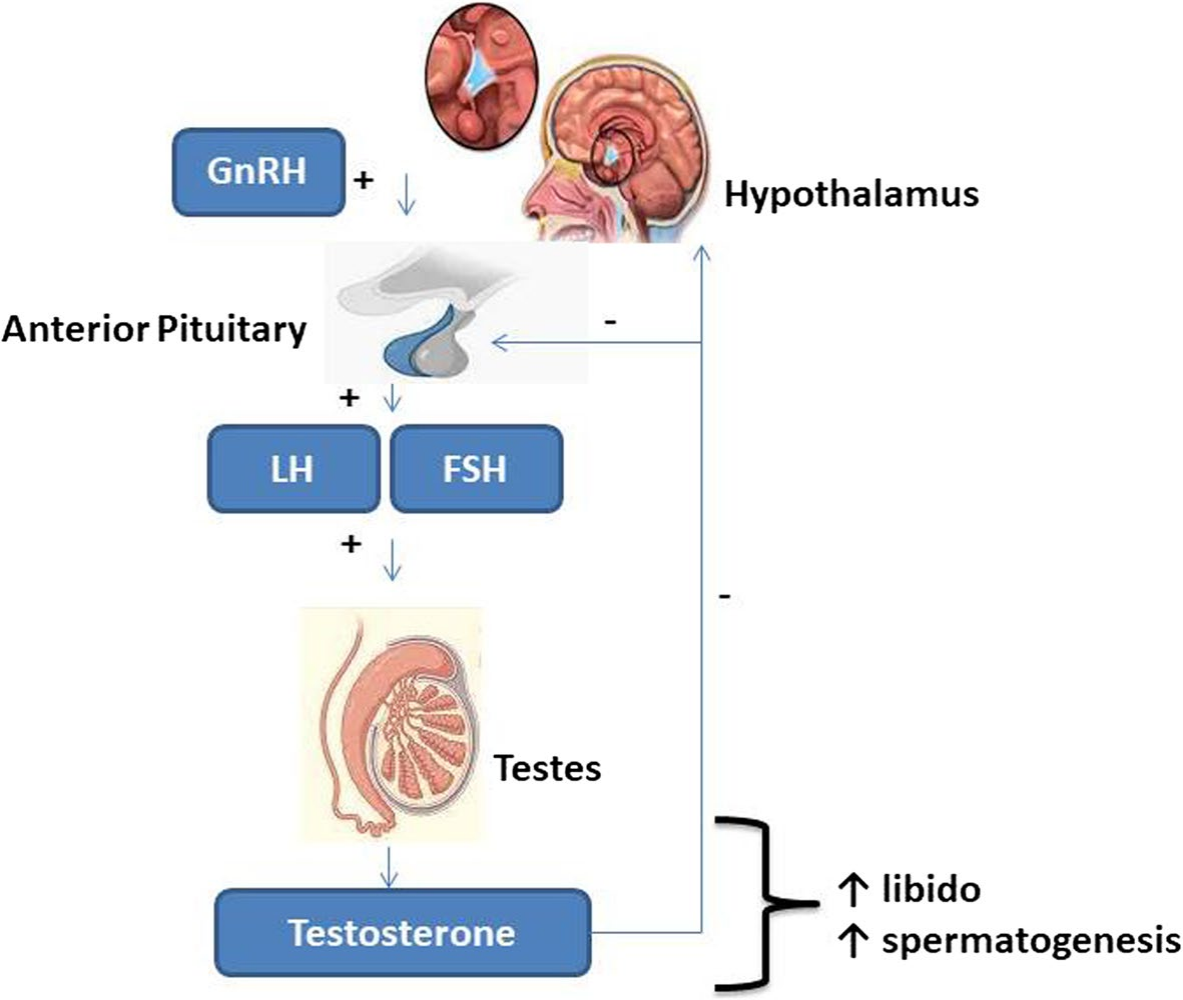
The hypothalamic-pituatary-testicular axis +  = stimulatory effect;—= inhibitory effeect. The hypothalamic-pituitary–testicular axis tightly regulates the male reproductive function. Gonadotropin releasing hormone (GnRH) is released in pulsatile manner from the hypothalamus to stimulate the release of the gonadotropins, which include the follicle stimulating hormone (FSH) and luteinizing hormone (LH). These gonadotropins stimulate the testes to maintain optimal testicular function. FSH stimulates the Sertoli cells to drive spermatogenesis, while LH stimulates the Leydig cells to promote testosterone biosynthesis, which is also required for sexual drive (libido) and spermatogenesis

Male reproductive health is as essential as general health since general health influences fertility and sperm quality directly or indirectly [91], which are influenced by the hypothalamic-pituitary–testicular (HPT) axis [5] (Fig. 1). Infertility is the inability to achieve conception after at least a year of adequate, unprotected sexual activity. Male factors alone or combined with female factors account for 30%–50% of infertility cases [73]. Sansone [97] reported that male infertility affects over 15% of all couples attempting to conceive, and in nearly half of these instances, male infertility is the primary or contributing issue. Male fertility decline is not a theoretical threat; research refers to a steady drop in sperm concentration over the last 35 years [95].

In examining any infertile male, a complete evaluation to identify predisposing factors is required [95]. Not unexpectedly, several acquired and congenital disorders may disrupt the delicate processes involved in spermatogenesis [95]. Age is substantially related to decreased sperm quality due to ongoing replication from altered spermatogonial stem cells [61]. Deoxyribonucleic acid (DNA) breakage and chromatin condensation may contribute to male infertility [101].

Increased reactive oxygen species (ROS) cause oxidative stress, the most well-known non-genetic cause of male infertility. ROS are required for capacitation, acrosome reaction, and eventually fertilization; nevertheless, both low clearance and excessive generation of ROS may impair sperm membrane integrity and cause DNA damage, resulting in decreased reproductive potential [69]. Fertile men's sperm has a greater antioxidant capacity than infertile men's sperm; also, immature teratozoospermia forms create more ROS than average, mature sperm [69]. Inflammatory processes and vascular illnesses, particularly varicocele, also promote ROS production [88].

Acquired testicular failure or reduced testicular function after testicular torsion, varicocele, orchitis, or cytotoxic therapy is often linked with azoospermia or oligozoospermia [48]. Azoospermia is often caused by genetic defects such as Klinefelter syndrome or microdeletions in the human male Y chromosomes AZF (azoospermia factor) region [62]. However, minor nucleotide polymorphisms are being explored as a potential "idiopathic" oligospermia source. Despite recent findings in the genetics of male infertility, most causes of oligozoospermia remain unclear [95].

Sperm deoxyribonucleic acid (DNA) damage may also be idiopathic [2]. It increases the frequency of sperm quality-related infertility [6]. Although the hypothalamic-pituitary–testicular axis influences spermatogenesis [29], other factors such as disease [2, 11, 14], heavy metal exposure [58, 59, 105, 6], and some medications, such as anti-psychotics, anti-depressants, and anti-convulsant, may impair male fertility. Hence, these medications are considered when evaluating male infertility [42].

### Effects of stimulants on testicular integrity and sperm quality

Methamphetamine is an illegal psychoactive drug that has been abused worldwide because of its stimulating and euphoric effects [80, 97]. In the realm of reproductive toxicology, methamphetamine has been identified as a critical substance [80]. Nudmamud-Thanoi and Thanoi [80] found that methamphetamine can change the shape, concentration, and activity of apoptotic cells in the seminiferous tubules of male rats.

A dose-dependent impact on sperm quality has been observed in rats treated with methamphetamine. Yamamoto et al. [104] found methamphetamine-induced apoptosis of seminiferous tubules in the male mouse testis 24 h after treatment with 5, 10, and 15 mg/kg of methamphetamine. This finding was corroborated by the reports of Alavi et al. [18], who observed increased apoptosis of the germ cells after repeated doses of methamphetamine. Methamphetamine-induced apoptosis of the germ cells may be due to its direct genotoxicity on the cells [80]. In addition, Alavi et al. [18] found that repeated administration of methamphetamine, particularly at 5 and 10 mg/kg, caused not just germ cell apoptosis but also a reduction in cell proliferation and an alteration of the proliferation/apoptosis ratio in the testis.

The hypothalamic-pituitary–testicular axis is not significantly impaired following persistent 3,4-Methyl​enedioxy​methamphetamine (MDMA) use [23]. The reports on the effect of MDMA on steroidogenesis in rat models are inconsistent [30]. Except for Harris et al. [53], who observed a marked rise in dehydroepiandrosterone levels but no impact on luteinizing hormone (LH) and follicle-stimulating hormone (FSH), there is a paucity of data reporting the impact of MDMA on male reproductive hormones.

Concerning histopathology indices, Barenys et al. [23] found that animals treated with MDMA had altered testicular tissue. Although the histoarchitecture of the epididymis was preserved following MDMA exposure, testicular histopathological examination revealed tubular degeneration and interstitial oedema.

The National Institute on Drug Abuse (2019) reported that cocaine addiction elicited epigenetic modification, resulting in an altered response to cocaine in an animal model's male but not female offspring. At a high concentration of cocaine, it binds specifically to testicular spermatozoa [76] and induces direct toxic effects. Prolong (≥ 5-year) cocaine usage was linked to reduced sperm concentration and motility and an increase in the proportion of sperm with aberrant morphology [25]. Short-term or long-term cocaine use slowed spermatogenesis and caused changes in the testes' ultrastructure [46, 90]. Cocaine stops spermatogenesis and tubule development immediately by causing cell death, sloughing, lipid droplets, and vacuoles. Under a light microscope, low-dose cocaine therapy decreased normal seminiferous tubules by 50% and high-dose by 40%. After moderate and high dosages, regressive tubules grew from 50 to 60% and from 60 to 90%, respectively, whereas normal tubules declined. At both cocaine doses, there was a significant (36% and 25% to 29%) decrease in the mean tubular diameters (MTD), excluding the tunica propria, and the surface occupied by the tubules (volume density, Vv, due to the sloughing of degenerating cells in the seminiferous tubules; both testes had a volume reduction [90]. Cocaine may have triggered apoptosis, which may explain these alterations [67].

Li et al. [66] found a statistically significant increase in germ cell death as early as 15 days after cocaine injection and lasting up to 90 days, consistent with the histology of cocaine-induced testicular shrinkage. These data imply that persistent cocaine treatment may promote apoptosis and kill germ cells in rat testes. TUNEL labelling showed rat seminiferous tubule apoptosis. TUNEL staining requires three DNA ends from apoptotic cells' DNA fragmentation. No testicular inflammation, high levels of necrotic cells, or random DNA breakage were seen after cocaine administration. Necrosis is unlikely to cause cocaine-induced germ cell loss. Cytochrome c and caspase cascade activation in cocaine-induced testicular apoptosis have been documented. Li et al. [67] reported cocaine-induced caspase-9 activations. Cocaine-treated testes showed higher caspase-3 activity on days 15–90 compared to controls. Cocaine exposure enhanced caspase-9 activity, which peaked on day 15 and decreased until day 90. At each time point, caspase-9 and -3 findings differ from controls. Available data reveals that two proteins in testicular tissue bind [3H] cocaine saturable and specifically. Both have different binding affinities, with one having a higher affinity than the other [65, 4, 72]. It is likely a step towards understanding the mechanism of action involved in cocaine-induced testicular apoptosis. Also, George et al. [46] reported that lowered fertility and smaller litter sizes were seen in rats exposed to continuous high doses of cocaine in males prior to mating.

### Effects of depressants on testicular integrity and sperm quality

In males, excessive alcohol intake may impair male reproductive function. Interestingly, reports on the effect of alcohol on male reproductive function are conflicting. Exposure to ethanol has been shown to change the hypothalamic-pituitary–gonadal axis, negatively affect the secretory function of Sertoli cells, and cause oxidative stress in the testes [10, 33, 40]. Long-term, excessive use of alcohol has been revealed to suppress circulating gonadotropin and testosterone, induce testicular shrinkage, and impair sperm production [47, 28]. However, an evaluation of about 8,000 men from the United States and Europe showed no change in the serum gonadotropin level but observed a linear rise in serum testosterone levels as alcohol consumption increased [60]. Findings for an alcohol impact on the testicular function in alcohol drinkers demonstrated that sperm parameter aberrations were related to considerably raised serum Follicle Stimulating Hormone (FSH), Luteinizing Hormone (LH), and 17-β-estradiol levels and dramatically reduced serum testosterone levels, indicating a primary testiculopathy. The serum prolactin level was normal [74].

Studies have also reported decreased sperm quality in heavy alcohol drinkers [50, 33], although Condorelli et al. [28] reported that there were no changes in sperm parameters as have been seen in males who consume alcohol regularly. Povey et al. [86] also reported no change in semen parameters following moderate alcohol use. In consonance with the findings of Povey et al. [86] and Condorelli et al. [28], some extensive cohort studies failed to find an association between male alcohol use and fecundity [52, 75, 106]. Also, alcohol use has been studied for its implications on testicular disease. Kuller and colleagues examined testis and liver pathology and estimated alcohol use in males who died abruptly from various causes. Twenty men (14%) exhibited a moderate-to-severe decline in spermatogenesis, while only nine exhibited significant or very significant liver fatty accumulation [64]. These data imply that alcohol affects testicular spermatogenesis more than liver tissue. It is generally recognized that alcohol usage causes considerable spermatozoal morphological alterations, including head breaking, middle distention, and tail curling [51]. Horak et al. [56] employed 32P-post labelling to measure bulky DNA adducts in sperm cells from 179 male donors and infertile patients. In this study, alcohol did not affect sperm DNA adducts [99]. Finally, Loft and his colleagues [68] assessed the degree of oxidative DNA damage measured by 7-hydro-8-oxo-20-deoxyguanosine (8-oxodG) in sperm DNA among 225 individuals planning their first pregnancy. The 8-oxodG level was not significantly correlated with alcohol usage.

Alcohol consumption and acute intoxication have been linked to sexual dysfunction, including problems with arousal and desire and erectile and ejaculatory dysfunction, all of which might contribute to male infertility [47, 28, 85]. Researchers are exploring new alcohol-damaging pathways. These pathways entail alcohol metabolism, induction of apoptosis, and hormone effects. Chronic alcohol consumption in male rats affects reproduction and offspring health [39].

Pajarinen & Karhunen [83] reported that in a prospective autopsy investigation, family and acquaintances of the dead provided extensive alcohol-use records to examine how alcohol affects spermatogenesis and testis morphometry. The autopsy cohort included 32 non-drinkers (daily consumption < 10 g) and 44 heavy drinkers (> 80 g). sp26 (81.3%) controls had normal spermatogenesis, while six (18.7%) had a partial spermatogenic arrest. Only 16 (36.4%) of heavy drinkers had normal spermatogenesis, 23 (52.3%) had a partial or full spermatogenic arrest, and five had Sertoli cell-only (SCO) syndrome. Heavy drinkers had a marginally reduced mean testicular weight compared to non-drinkers. The testicular weight was somewhat lower in controls and heavy drinkers with spermatogenic arrest and considerably lower in heavy drinkers with SCO syndrome compared to males with normal spermatogenesis.

The disparity observed in the reported human and experimental studies on the impact of alcohol on testicular and sperm integrity might be due to the different study designs. According to Rehm [89], most human studies use self-reported data collected through questionnaires. It is subject to recall bias. Also, the amount of alcohol consumed was not objectively quantified.

Studies have shown that despite the ban on Rohypnol in many countries, including Nigeria, it is a leading substance of abuse [19, 45] with a propensity for dependence [44]. Hayam et al. [54] examine how rohypnol affects developing testes. Sixteen pregnant rats' offspring were utilized. Four groups of pregnant rats were evenly split. Controls were the first group's offspring. The second group treated female offspring. This group of pregnant rats received a single oral therapeutic dosage of 0.036 mg of rohypnol daily from conception through the first 10 days after birth. From the 10th to the 30th day postnatally, their children got 0.0036 mg of rohypnol orally. The third group consisted of the treated offspring of untreated females. This group's pregnant females were untreated, but their offspring were treated like the second group. The 4th group consisted of non-treated offspring of treated females. Similar to the 2nd group, this group's pregnant rats were treated, but their pups were not. All groups' offspring's testes were obtained at day 30 postnatally. All of the groups that were given Rohypnol had slower spermatogenesis. The short seminiferous tubules without a central lumen, the three to four rows of seminiferous cells, the absence of early spermatids almost entirely, and the slow transition of supporting cells into Sertoli cells all demonstrated this. Histopathological effects were also notable. In the 2nd group's testis, numerous seminiferous cells were heavily discoloured, undetectable, and degraded. The third and fourth groups had mildly impacted seminiferous cells, respectively. All groups had significant Leydig interstitial cell damage. They were small collections in the 4th group or distributed between the second and third groups' seminiferous tubules. The 2nd group's testis showed that spermatogonia were somewhat impacted, supporting cells were abundant and moderately affected, and primary spermatocytes and Leydig interstitial cells were the most affected. Oluwole and his colleagues (2021) reported that rohypnol impaired sexual urge and sexual activity by suppressing the hypothalamic-pituitary–testicular axis in an animal model [81]. Using rohypnol led to longer mount, intromission, and ejaculatory latencies, as well as lower ejaculatory frequencies. It also resulted in a significantly extended postejaculatory interval and a lower sperm count, motility, and viability, but an increase in the proportion of aberrant sperm morphology [81].

### Effects of narcotics/opioids on testicular integrity and sperm quality

Morphine and morphine-like opioids have been used for many years to make people feel "high" or "mellow" [27]. One of the most frequently misused opioids in the United States is codeine [22, 45], and it is frequently linked to the onset of drug abuse [45]. Although codeine has been shown to enhance libido and male sexual activity, it may cause a marked reduction in copulatory efficiency and fertility indices [7]. Chronic codeine use has also been shown to cause testicular degeneration, as evidenced by vascular congestion, vacuolation, germ cell loss, and arrest of germ cell maturation [12, 77], as well as circulating testosterone suppression via upregulation of oxidative stress-sensitive caspase 3 signalling [12], and downregulation of the human epidermal growth factor receptor 2/Antigen KI-67 (HER2/Ki67) pathway and modulation of tumor protein p53/ B-cell lymphoma-2 (p53/Bcl-2) signaling [8]. In addition, codeine lowers sperm quality and induces oxidative sperm DNA damage and apoptosis [5]. Codeine may also exert an epigenetic effect as it has been reported to impair testicular and sperm DNA integrity in male offspring birthed by codeine-exposed dams via reprogramming testicular cytoprotective and spermatogenic genes and steroidogenic proteins [13]. Tramadol has been proven to severely lower sperm count, viability, and normal morphology [79]. Azari et al. [20] demonstrated that tramadol might significantly reduce sperm concentration, motility, and vitality at 10 and 20 mg/kg body weight. The effect of tramadol on the sperm quality of male albino rats was also shown to be substantial at 50 and 100 mg/kg body weight, according to Esua et al. [41].

In a separate study, Ahmed & Kurkar [3] evaluated tramadol's effects on male adult rats' testicles. Twenty albino adult male rats comprised the control and tramadol groups. Tramadol was subcutaneously administered to rats three times a week for eight weeks. Tramadol raised prolactin and estradiol while decreasing luteinizing hormone (LH), follicle-stimulating hormone (FSH), testosterone, and total cholesterol. Tramadol also boosted testicular nitric oxide, lipid peroxidation, and antioxidant enzyme activity. Tramadol decreased primary spermatocytes, rounded spermatids, Leydig cells, and sperm count. Immunohistochemistry showed that tramadol enhanced testicular endothelial nitric oxide synthase. In addition, studies have shown that tramadol distorts the seminiferous tubules and reduces spermatogenic cells [37, 93]. Tramadol and morphine have been shown to cause structural anomalies and distort the normal rat testis histological structure [102, 98, 105]. Abdellatief and his colleagues [1] studied the effects of chronic tramadol administration on gonadotrophic and sex hormones and histological and morphometrical alterations in rat testicular tissue. Tramadol was administered alone to mature male albino rats. After 30 days of treatment, tramadol lowered luteinizing hormone (LH), follicle-stimulating hormone (FSH), and testosterone. They observed degenerative changes in seminiferous tubules in their study. The spermatogenic layers were contracted and separated, with the tubular foundation membrane disorganized and vacuolized. The morphometric study showed a considerable reduction in tubular diameter and epithelial height. Apoptotic cells and abnormal ultrastructure were seen in all spermatogenic lineage cells. Sertoli cell connections, vacuolation, and large lipid droplets were seen. Leydig cells have euchromatic nuclei and an expanded endoplasmic reticulum. Ibrahim & Salah-Eldin [57] also corroborated these findings with an increased apoptotic index. Elevated B-cell lymphoma-2 (Bcl-2) levels related to X protein and caspase-3 expression were associated with a significant drop in anti-apoptotic sBcl-2 in tramadol-treated male adult albino rats.

### Effects of hallucinogens on testicular integrity and sperm quality

Although researchers have studied ketamine extensively, little is known about the long-term effects of other hallucinogens on male fertility. Chronic use of ketamine reduces the weight of male reproductive organs [87, 84]. Qi et al. [87] and El Shehaby et al. [36] reported that ketamine upregulated testicular apoptosis and impaired spermatogenesis, evidenced by a significant reduction in the mean Johnsen score. Ketamine has also been revealed to disrupt the seminiferous tubular structure and reduce germ and seminiferous luminal sperm cells [87]. Tramadol-treated male Wistar rats' testicular tissues showed varied and patchy histopathological alterations, according to Paulis et al. [84]. Vascular congestion and a change in the shape of the seminiferous tubules were caused by a broken basement membrane, underdeveloped germ cells, desquamation of the germ cells, and swelling of the interstitium (oedema). The most significant histological observation was the decrease in normal spermatogenic cells and spermatozoa in several tubules. Despite enormous germ cell loss, Sertoli cell numbers did not decrease, and vimentin expression increased dramatically compared to the control group.

Tan et al. [100] reported that ketamine administration induced a marked decrease in sperm count and motility and a corresponding increase in abnormal sperm cells. These effects were reversed and approached normalcy 4 weeks after the cessation of ketamine administration.

Qi and colleagues [87] found that the messenger RNA (mRNA) expression of gonadotropin-releasing hormone (GnRH) was considerably reduced in the ketamine group when compared to the control group. Qi et al. [87] observed that ketamine reduced circulating LH, FSH, and testosterone. The reduced testosterone levels in ketamine-treated rats may be due to decreased gonadotropins and ketamine-led suppression of Leydig cell function [84, 87]. Ketamine may cause lipid peroxidation and apoptosis of the Leydig cell [103]. El Shehaby et al. [36] demonstrated that ketamine impaired testicular and erectile function. It was coupled with reduced pre-coital sexual behaviour and ejaculation. The histological study turned up evidence of significant dysspermatogenesis [34]. A positive correlation exists between serum testosterone and catalase, and a positive correlation between luteinizing hormone (LH) and total antioxidant capacity in serum (TAC) supports it [35].

### Effects of cannabis/marijuana on testicular integrity and sperm quality

Cannabis sativa, the plant from which marijuana is made, is a mind-altering (psychoactive) substance. The chemical composition of marijuana is staggering, with over 480 different components. THC (delta-9-tetrahydrocannabinol) is widely regarded as the primary component responsible for the psychoactive effects of cannabis.

Pagotto et al. [82] reported that cannabinoids lower LH, reduce testosterone synthesis and release, and inhibit spermatogenesis. Cannabis has been demonstrated to induce gonadotoxicity by triggering oxidative stress [71, 16]. It is associated with suppressing circulating LH, FSH, and testosterone [38, 17] and reduced sperm quality [49, 16].

Alagbonsi and Olayaki [15] revealed that 9-tetrahydrocannabinol reduced sperm motility, average path velocity (VAP), curvilinear velocity (VCL), straight-line velocity (VSL), the amplitude of lateral head displacement (ALH), and beat cross frequency (BCF). Gundersen et al. [49] found that men who smoked marijuana more than once a week had lower sperm concentration, total sperm count, percentage of motile sperm, and percentage of morphologically normal forms. Surprisingly, Hehemann et al. [55] found that although marijuana may also have a negative impact on sperm quality, notably morphology and volume, it may protect against aberrant sperm motility.

Testicular atrophy in animal models has been linked to the use of cannabis [21]. A decrease in spermatogonia, corresponding basement membrane damage with relatively scanty cytoplasm and shrunken nuclei, and a reduction in seminiferous tubule diameter were observed in rat models of cannabis use [71, 94, 16].

Belladelli et al. [24] performed a meta-analysis. Their findings showed that cannabis research on reproductive and sexual health is poor. Cannabis usage has no clinical impact on testicular function, according to their comprehensive study and meta-analysis. Due to the limited number of studies and the variability of the available research, they could not rule out a cannabis influence on testicular function, and the present study does not ensure safety. There are some drawbacks. First, most research did not disclose cannabis use profiles, which restricts the data's interpretability since cannabis use frequency and amount vary [26]. The dose-dependent impact of cannabis consumption was also impossible to study due to its unpredictability. While they employed a classification of semen quality per World Health Organization (WHO) reference levels, the researchers could not ignore the potential for a difference between cannabis users and non-users if they assessed the actual values of semen parameters. Pregnancy results are essential to defining cannabis's most important reproductive clinical aims. Recruitment tactics may prejudice against age or geography (e.g., markets versus fertility clinics). Finally, all research used self-reported cannabis usage, which may be unreliable due to stigma or fear of penalties. However, recent research supports survey methods [96].

## Conclusion and recommendations

**Fig. 2:**
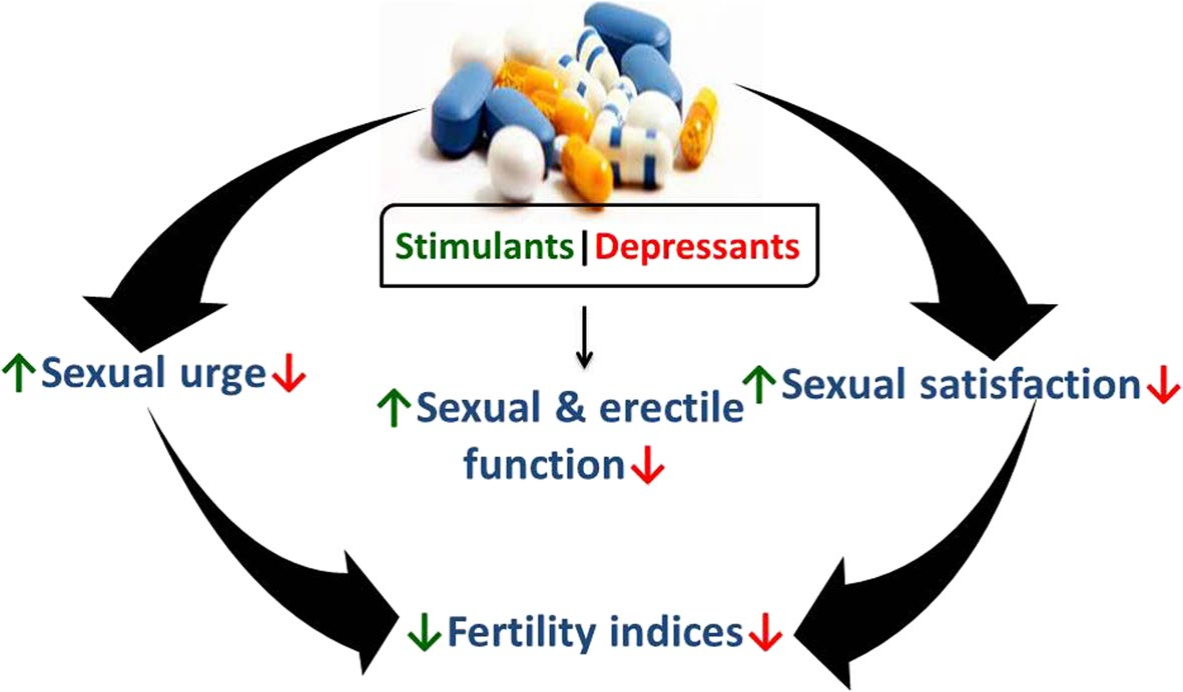
Effects of psychoactive drugs on sexual function and fertility indices. Psychoactive drugs usually act as stimulants or depressants irrespective of their classes. Depressants have been reported to suppress (red arrow) sexual urge, sexual and erectile function, and sexual satisfaction via downregulation of circulating androgen and stimulatory neuroendocrine like dopamine, resulting in reduced fertility indices. On the other hand, although stimulants may elicit increased (green arrow) sexual urge, sexual and erectile function, and sexual satisfactory via a testosterone-independent signaling, they also induce reduced fertility indices

**Fig. 3:**
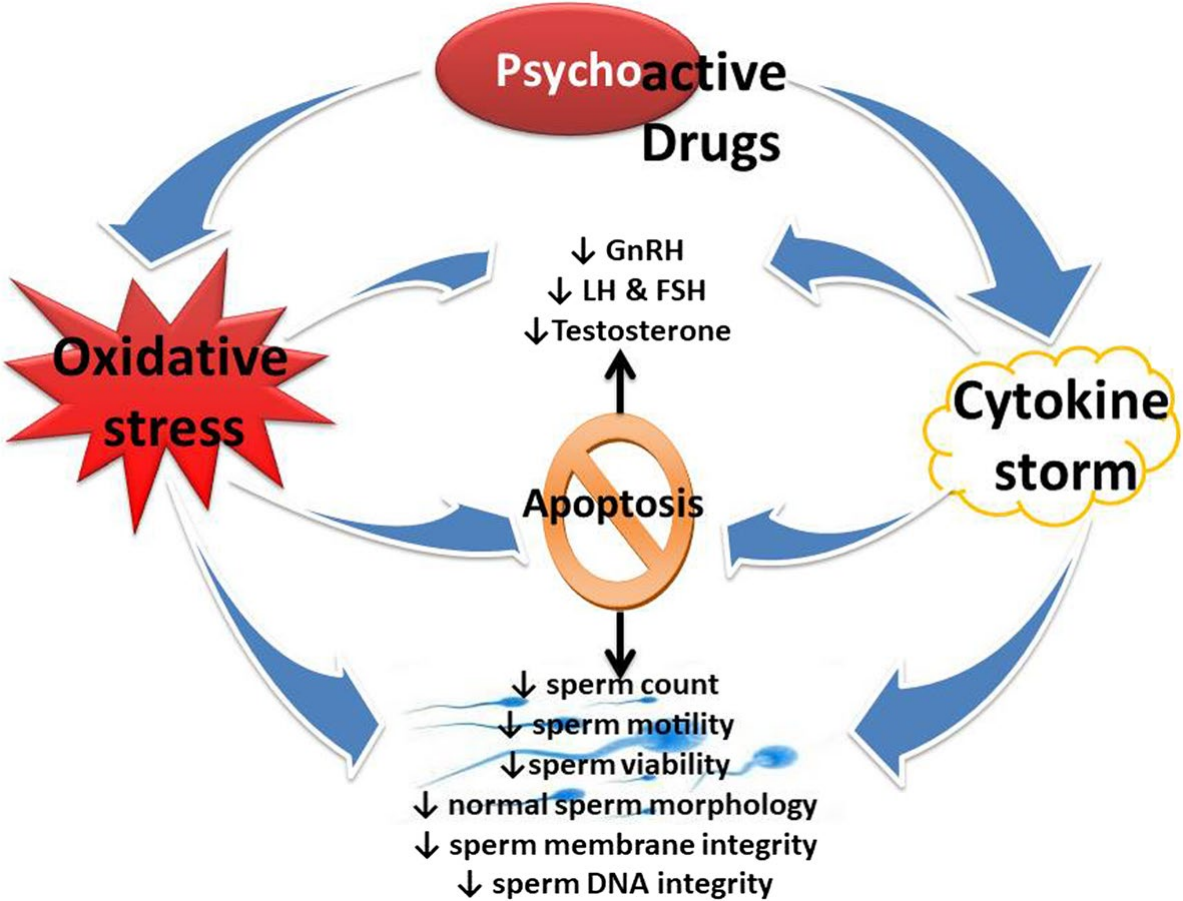
Effects of psychoactive drugs on testicular integrity and function. Psychoactive drugs promote increased generation of reactive oxygen species (ROS) in the testes that overwhelms the scavenging capacity of the protective testicular antioxidant system, leading to oxidative stress. These drugs also increase the accumulation of pro-inflammatory cytokines, resulting in cytokine storms. Testicular oxidative stress could be a cause and/or a consequence of the cytokine storm; this leads to a vicious cycle of oxido-inflammatory state that disrupts hypothalamic-pituitary–testicular axis and sperm integrity, resulting in altered testicular function viz. downregulation of the release of gonadotropins and testicular testosterone biosynthesis, and impaired spermatogenesis and reduced sperm quality

**Fig. 4:**
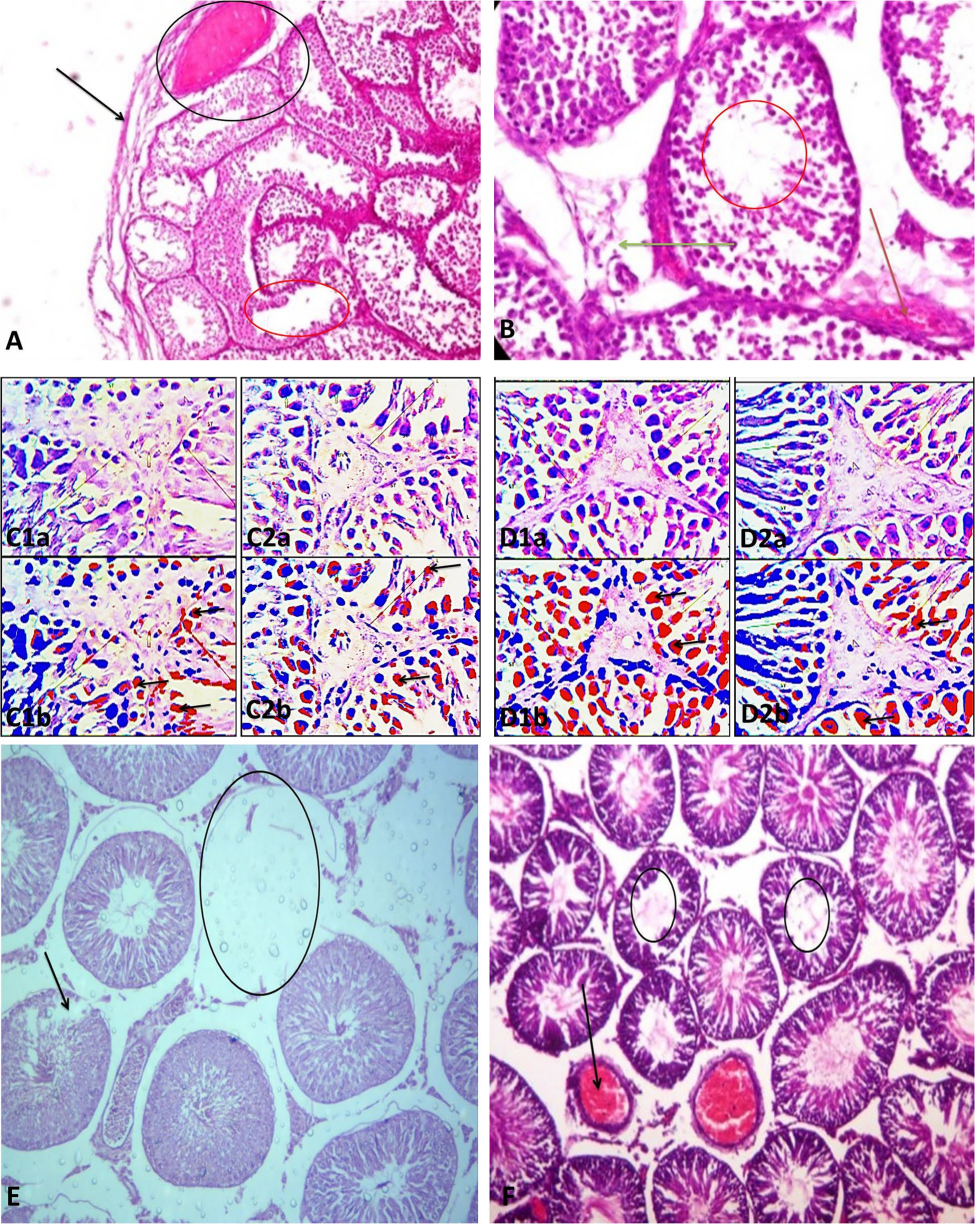
Effect of selected psychoactive drugs on testicular histoarchitecture. A-B) Codeine-treated rabbits showed distorted testicular architecture. The seminiferous tubules showed thickened propria indicative of cessation of spermatogenesis (black arrow). There are vacuolation, sloughed germ cells, maturation arrest, and reduced mature sperm cells within the tubular lumen (red circle). There is evidence of vascular congestion (black circle and red arrow). The leydig cells appear reduced (green arrow). (Photomicrographs are from our laboratory-published: [12]. Plate C1a and 1b are the original and pseudo images respectively of the testicular histoarchitecture of vehicle-treated control rats compared with those of codeine-treated rats (C2a and 2b) showing p53 expression. Codeine treatment led to significantly increased p53 expression. Also, plate D1a and 1b are the original and pseudo images respectively of the testicular histoarchitecture of vehicle-treated control rats compared with those of codeine-treated rats (C2a and 2b) showing Bcl-2 expression. Codeine treatment led to marked reduction in Bcl-2 expression. These findings are suggestive of codeine-induced apoptosis. (Photomicrographs are from our laboratory-published: [8]. E) Rohypnol treatment led to degeneration of seminiferous tubules (black circle) and germ cells (black arrow), with widened interstitial space. (Photomicrograph is from our laboratory-In Press, [9]. F) Methamphetamine caused degeneration of germ cells and reduced mature sperm cells in the tubular lumen (black circle). It also led to vascular congestion within the interstitial space (black arrow). (Photomicrograph is from our laboratory-unpublished)

Summing up, psychoactive drugs exert negative effects on male reproductive functions (Table 2), viz., sexual urge (Fig. 2), androgen synthesis, spermatogenesis, and sperm quality (Fig. 3). These drugs directly induce testicular toxicity by promoting ROS-dependent testicular and sperm oxidative damage, inflammation, and apoptosis (Fig. 4), and they also suppress the hypothalamic-pituitary–testicular axis. This results in the suppression of circulating androgen, impaired spermatogenesis, and reduced sperm quality.

**Table 2:** Effects of psychoactive drugs on male fertility

Drugs	Effect	References
**Stimulants**		
Methamphetamine	Suppression of circulating testosterone, distortion of testicular histoarchitecture (tubular degeneration and interstitial edema), apoptosis of germ cells	[18, 23, 53, 80, 104]
Coccaine	Epigenetic modification	[78]
**Depressants**		
Alcohol	Suppression of hypothalamic-pituitary–testicular axis, sexual dysfunction, impairment of testosterone biosynthesis and spermatogenesis, testicular shrinkage	[10, 28, 33, 40, 47, 85]
Rohypnol	Suppression of hypothalamic-pituitary–testicular axis, sexual dysfunction, reduced sperm quality	[81]
**Opioids/narcotics**		
Codeine	Suppression of hypothalamic-pituitary–testicular axis, reduced circulating testosterone, testicular and sperm oxidative damage, testicular cytoprotective and spermatogenic genes reprogramming	[5, 7, 8, 12, 13, 77]
Morphine	Distortion of testicular histoarchitecture	[98, 102]
Tramadol	Reduced sperm quality, distortion of testicular histoarchitecture	[20, 37, 41, 79, 93]
**Hallucinogens**		
Ketamine	Reduced testicular weight and distortion of testicular histoarchitecture, impairment of spermatogenesis, reduced sperm quality, reduced circulating testosterone, impaired male sexual behaviour	[36, 84, 87, 100]
**Cannabis**	Reduced circulating gonadotropins and testosterone, testicular damage, reduced sperm quality	[16, 17, 71, 94]

## Data Availability

Not applicable.
